# Biocompatible Mesoporous Silica–Polydopamine Nanocomplexes as MR/Fluorescence Imaging Agent for Light-Activated Photothermal–Photodynamic Cancer Therapy *In Vivo*

**DOI:** 10.3389/fbioe.2021.752982

**Published:** 2021-11-09

**Authors:** Jiahui Lu, Chen Ni, Jie Huang, Yawen Liu, Yingkai Tao, Pengcheng Hu, Yong Wang, Shaohui Zheng, Meilin Shi

**Affiliations:** ^1^ School of Medical Imaging, Xuzhou Medical University, Xuzhou, China; ^2^ School of Medical Imaging, Hangzhou Medical College, Hangzhou, China; ^3^ Department of Dermatology and Venereal Diseases, The First People’s Hospital of Changzhou, Changzhou, China; ^4^ Institute of Medical Imaging and Digital Medicine, Xuzhou Medical University, Xuzhou, China

**Keywords:** chlorin e6, magnetic resonance imaging, fluorescence imaging (FI), photothermal therapy, photodynamic theraphy, core shell nanoparticles

## Abstract

Conventional cancer phototherapy with single modality suffers from low therapeutic efficacy and undesired posttreatment damage for adjacent normal tissues. Therefore, the lower NIR laser irradiation power is vital to the reduction or preclusion of risk of scalds and burns in normal tissues. Herein, we rationally proposed a novel multifunctional nanocomplex, which enabled good magnetic resonance (MR) imaging contrast effect and promising photothermal conversion efficacy. The prepared core/shell nanocomplexes [MSN-Ce6@PDA (Mn)] were composed of chlorin e6-embedded mesoporous silica/nanoparticle composites as the cores, and then polydopamine and manganese ions were conjugated on the cores to form protective shells. The MSN-Ce6@PDA (Mn) nanocomplexes revealed superior properties in colloidal stability, photothermal conversion, reaction oxygen species generation, magnetic resonance imaging, etc. Under the guidance of MR and fluorescence imaging, these MSN-Ce6@PDA (Mn) nanocomplexes were found to be primarily accumulated in the MDA-MB-231 tumor area. Furthermore, the combined photodynamic and photothermal therapy exhibited strong inhibition to the growth of MDA-MB-231 tumor *in vitro* and *in vivo*. Besides, the MSN-Ce6@PDA (Mn) nanocomplexes also exhibited excellent biocompatibility and low damage to the healthy animals. Hence, the results demonstrated that the prepared MSN-Ce6@PDA (Mn) nanocomplex would be a promising potential for multimodal imaging-guided phototherapy.

## Introduction

The application of nanomaterials in biomedicine facilitates the integration of multiple modalities into an individual platform, which exhibits high effectiveness to advance cancer diagnosis and therapeutics (Goel et al., 2018; Kang et al., 2020). In order to overcome the weakness of mono-therapy, notably, inefficiency and inefficacy, frequent recurrence, and severe adverse effects (Liu S. et al., 2019; Wang et al., 2020; Liu et al., 2021), a large number of sophisticated nanostructures were prepared with multiple treatment regimens. Noteworthily, light-mediated photo-therapeutic approaches, such as photothermal therapy (PTT) and photodynamic therapy (PDT), have provided great merits fairly of noninvasiveness and minimal injury to normal tissues in cancer treatment. With the photoactivation of photosensitizers in the tumor region, the therapeutic principle of PDT could be produced by the generation of cytotoxic oxygen species. On the other hand, PTT relied on the local hyperthermia generated by the photo-absorbing materials under NIR irradiation to cause tumor cell apoptosis and death (Yu et al., 2019; Sun et al., 2020; Yi et al., 2020). Currently, PDT has exhibited good therapeutic outcome in the treatment of various cancers, such as breast cancer (Banerjee et al., 2017), melanoma (Baldea et al., 2012; Chen et al., 2021), gynecological cancers (Allison et al., 2005), and cholangiocarcinoma (Lee et al., 2012). Furthermore, some formulations have been also evaluated in the clinical stage. However, as for PTT, not so many formulations went to clinical study despite their superior therapeutic outcome in the thermal ablation of tumor tissues in animal studies (Li et al., 2020). Therefore, the combination treatment of PDT with other therapeutic strategies such as PDT–PTT, PDT–chemotherapy, and immunotherapy may be applied in the clinical scenario.

Recently, PTT/PDT-combined therapy has been applied as a positive therapeutic strategy, which has garnered a high therapeutic index (Guan et al., 2019; Hu et al., 2019). Nevertheless, most PDT photosensitizers absorb light in the visible range, resulting in limited penetration depth (Fan et al., 2021). In contrast, PTT, which utilizes the photosensitizer excited by near-infrared red light, has improved the penetration depth (Zhou et al., 2021). More importantly, PTT has been reportedly validated to produce a good short-term effect (Wen et al., 2021; Mengya et al., 2021). Therefore, the combination of PTT and PDT represents a potential of relatively noninvasive modality for the therapy of superficial tumor, such as melanoma and breast cancer (Ma et al., 2020; Guoyun et al., 2020). High-powered laser irradiation would rapidly increase the temperature of the irradiated area. Nonetheless, the excessive heat therein would destroy both tumor tissue and the adjacent healthy tissues. To minimize the risks of damage to the healthy tissues, a nanoplatform which can exert satisfactory therapeutic effects under low-powered laser irradiation is more preferable (Szezerbaty et al., 2018; Wan et al., 2020; Ni et al., 2020).

Chlorin e6 (Ce6) is a photosensitizer approved by the FDA for clinical application in 2004. This photodynamic agent has facilitated the efficient PDT and fluorescence imaging (Wei et al., 2018; Sun et al., 2019). However, the poor aqueous solubility of Ce6 caused the inclination of agglomeration resulting in poor performance on cellular uptake. Therefore, it is of great significance to improve its solubility or colloidality for the application in biomedicine (Nafiujjaman et al., 2016; Amirshaghaghi et al., 2019).

To overcome the above drawbacks, the core–shell structure provided an excellent strategy to formulate Ce6 inside a nanoplatform for further biomedical application (Ai et al., 2015; Liu P. et al., 2019; Ri et al., 2020). Mesoporous silica is highly biocompatible, with a great surface area and adjustable pore sizes, which are extensively applicable in the Ce6 formulation (Croissant et al., 2018; Manzano and Vallet-Regí, 2020). In addition, the advent of polydopamine (PDA) as a surface coating brings prospect to enhance hydrophilicity properties and biocompatibility. Furthermore, PDA could also be used as a photothermal agent in tumor ablation owing to its high NIR absorbance to realize satisfactory photothermal conversion efficiency. A more attractive property for PDA modification lies in its chemical reactivity, such as the ion-coordinating ability (e.g., Gd^3+^, Mn^2+^, Fe^3+^), which enable T_1_/T_2_-weighted MRI agents (Lee et al., 2007; Liu et al., 2013; Hu et al., 2016; Liang et al., 2020; Lu et al., 2021).

**SCHEME 1:**
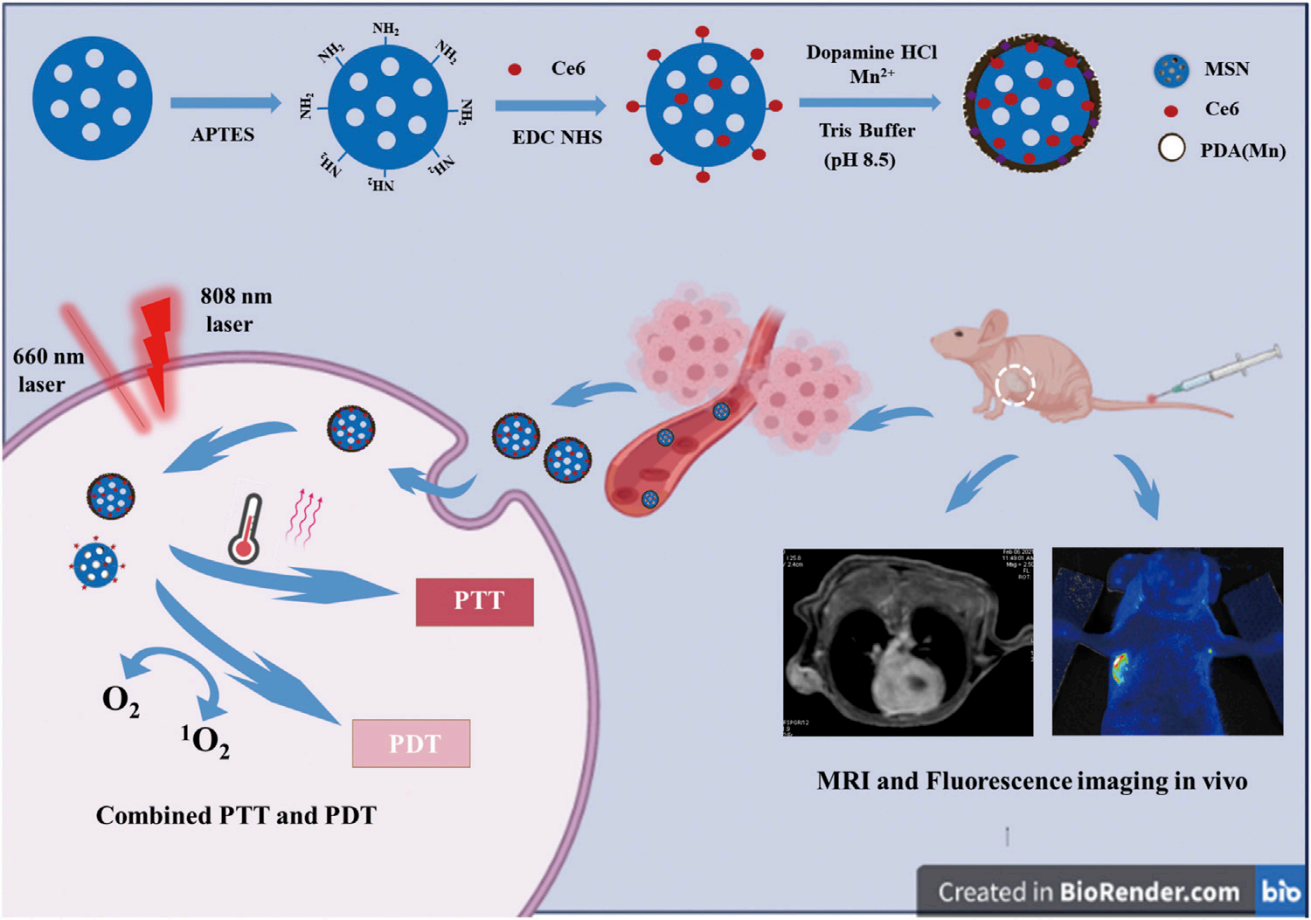
Scheme for the preparation process and working mechanism of MSN-Ce6@PDA (Mn) (MCPM NPs) for MRI and fluorescence imaging and photothermal and photodynamic combination regimen.

In our study, we proposed core–shell nanocomplexes for synergistic PDT–PTT therapy, as illustrated in Scheme 1. Nanoscaled MSN-conjugating Ce6 on the surface was prepared as a core with modification of the PDA shell to improve hydrophilicity and Mn^2+^ incardination to facilitate MR imaging and PTT. The resulted mesoporous silica@polydopamine core–shell nanocomplexes enabled the stable delivery of Ce6 and achieved magnetic resonance/fluorescence imaging and photothermal–photodynamic synergistic therapy for breast cancer.

## Experimental Procedures

### Materials

The chemicals involved in this study were shown as follows: cetyltrimethylammonium chloride (CTAC), manganese chloride tetrahydrate, triethylamine (MnCl_2_·4H_2_O), anhydrous toluene, cyclohexane, and tetraethoxysilane (TEOS) (Sinopharm Chemical Reagent Co. Ltd., Shanghai, China); TEOS and dopamine hydrochloride (Aladdin, Shanghai, China); and Ce6 (Frontiersci, Logan, UT, USA).

### Synthesis of MSN-Ce6@PDA (Mn)

#### Preparation of Ammonization of Mesoporous Silica (MSN-NH_2_)

Mesoporous silica nanoparticles were synthesized and functionalized as previously described (Lee et al., 2007; Shen et al., 2014). Briefly, CTAC (5 g) and TEA (0.09 g) were prepared in aqueous solution (18 ml) with continuous agitation at 70°C for 1 h. Thereafter, a mixture of cyclohexane and TEOS (20 ml, volume ratio of 4:1) was slowly added into the solution and kept at 70°C for 24 h. Afterward, the deposit was harvested *via* centrifugation and rinsed thrice with ethanol. Subsequently, the product underwent removal of the CTAC template in triplicate with 1 wt% sodium chloride–anhydrous methanol solution, followed by freeze-drying of the prepared mesoporous silica nanoparticles.

The mesoporous silica nanoparticles were aminated by the toluene reflux method (Ebrahimi-Gatkash et al., 2017). Firstly, the mesoporous silica powder (100 mg) was dispersed into 10 ml of anhydrous toluene. With the solution heated to 70°C, 15 μl aminopropyl-triethoxysilane was added into the dispersion for further reaction for 4 h under a nitrogen atmosphere. Thereafter, the solution was centrifuged to isolate the final product and was further rinsed thrice in deionized water. Finally, the prepared nanomaterial of mesoporous silica (MSN-NH_2_) was freeze-dried.

#### Synthesis of Ce6-Conjugated Mesoporous Silica (MSN-Ce6)

The covalent binding of Ce6 to MSN-NH_2_ was conducted by the standard 1-(3-dimethylaminopropyl)-3-ethylcarbodiimide (EDC)–N-hydroxysuccinimide (NHS) reaction (Wang et al., 2019). Briefly, Ce6 (3 mg) was activated by EDC–NHS (mass ratio of 1:1.2) solution. Then, the above mixture was stirred for 15 min at 120 rpm and 37°C. Following activation, MSN–NH_2_ (6 mg) was added and kept at 37°C for 2 h. The product was purified by repeated centrifugation, water replacement, and rinsing until the supernatant becomes colorless.

### Synthesis of MSN-Ce6@PDA (Mn)

The prepared MSN-Ce6 was mixed with dopamine hydrochloride (3 mg) and thereafter added into Tris solution (2.5 ml, pH 8.5) and stirred overnight. The mixture was chelated into MnCl_2_·4H_2_O solution for agitation for 4 h at 40°C. Subsequently, with the mixture rinsed in deionized water and centrifuged thrice (13,000 rpm, 30 min, 4°C). MSN-Ce6@PDA (Mn) (monocalcium phosphate monohydrate nanoparticles (MCPM NPs)) obtained were freeze-dried and stored at 4°C for subsequent experimentation.

### Characterization

The prepared MCPM NPs were physiochemically characterized as the following aspects. With respect to morphology and structure, the nanoparticles were imaged by transmission electron microscopy (TEM, FEI Tecnai 20) and a field emission scanning electron microscopy (Tescan MAIAA3). The Fourier transform infrared (FT-IR) analysis was conducted on a Piketech FTIR spectrometer. The UV-vis absorbance spectra were obtained on NanoDrop, and the fluorescence imaging excitation spectra were acquired on a PerkinElmer spectrophotometer. The zeta potential and size distribution measurements were performed on a Malvern Zetasizer Nano ZS system. The nitrogen adsorption curves were tested on a physical adsorption meter (ASAP 2460). The energy-dispersive spectroscopy (EDS) and X-ray photoelectron spectroscopy (XPS) spectra were determined on an X-ray photoelectron spectrometer (Thermo ESCALAB 250Xi). As for MR relaxivity assessment, several samples of MCPM NP solutions were prepared with an escalation of Mn concentrations in PBS (0, 0.0244, 0.0489, 0.0897, 0.1783, 0.3566, 0.7132 mM). T_1_-weighted phantom images and T_1_ relaxation duration were obtained with a 3.0-T GE Discovery 750 W RM system. The r_1_ relaxivity was thus calculated from the linear slope obtained under a spin-echo sequence and the following parameters: TR = 425 ms, TE = min full, TI = 28.0, matrix size = 328 × 224, field of view = 24 cm × 24 cm, slice thickness = 3.0 mm, slice gap = 1.5 mm.

### Photothermal and Singlet Oxygen Generation Performance

The photothermal conversion of MCPM NPs was evaluated *via* a continuous-wave fiber-coupled diode laser (808 nm, FC-808-10W, Changchun New Industries Optoelectronics Technology Co. Ltd., Changchun, China). Aqueous dispersions of MCPM NPs (1 ml) at several concentrations (0, 0.03, 0.1, 0.3, 0.5 mg ml^−1^) were loaded in a quartz cuvette and irradiated with an 808-nm laser (1 W cm^−1^) for 10 min. In parallel, the exposure of MCPM NPs (0.3 mg ml^−1^) to the 808-nm laser was at graded power densities (0.25, 0.5, 1, 1.5 W cm^−1^), with the temperature range recorded with a FLIR thermal camera at an interval of 30 s. As for the photostability and photothermal stability, MCPM NP dispersion (0.3 mg ml^−1^) also underwent exposure to the 808-nm laser (1 W cm^−1^). Thereafter, the hydrodynamic sizes of MCPM NPs with or without laser irradiation were measured in a 7-day period. In addition, with the 808-nm laser powered on and off, the temperature of samples gradually declined and restored. For the evaluation of the photothermal stability of MCPM NPs, eight on/off cycles were conducted with the laser turned on and off interchangeably.

The singlet oxygen behavior of MCPM NPs was assessed *via* a continuous-wave laser (660 nm, Wuhan Boji Century Technology Co., Ltd., Wuhan, China). The irradiation power was 100 mW cm^−2^ (10 min), and the 1,3-diphenylisobenzofuran (DPBF) dye was employed for the detection of singlet oxygen. In the typical experiment, 30 μl of the DPBF (10 mM) was added to the MCPM NPs (0.3 mg ml^−1^, 0.1 ml). The aqueous dispersion of MCPM NPs (2 ml) was loaded in a quartz cuvette and treated with different lasers (no laser, 808 nm laser, 660 nm laser, 808/660 nm laser). The fluorescence absorbance value of the remaining DPBF was detected by a microplate reader (Thermo Scientific, Waltham, MA, USA). Over the course of 10 min of light exposure, we collected values every minute.

### Evaluation of *in Vitro* Biocompatibility and Cytotoxicity

The *in vitro* biocompatibility tests were performed using Cell Counting Kit 8 (CCK-8) assay on MDA-MB-231 human breast cancer cells and NIH3T3 mouse embryonic fibroblast cells provided by the Cell Bank of the Chinese Academy of Sciences (Shanghai, China).

Briefly, MDA-MB-231 and NIH3T3 cells were seeded into 96-well plates (5 × 10^3^ cell/well) for 24 h. After coculture with MCPM NPs at a gradient of concentrations (0–0.5 mg ml^-1^) for 24 h, 10 μl fresh CCK-8 solution was added for further incubation of 2 h. The absorbance of the MPCP NP solution was determined using a microplate reader at 450 nm. Identical to the manipulation of cell viability assay, the relative cell viability post irradiation was also determined with the standard CCK-8 method.

Subsequently, we also explored the photothermal–photodynamic therapeutic effect on MDA-MB-231 tumor cells. Following overnight culture in 96-wells, MDA-MB-231 cells were treated with MCPM NPs at the end of laser irradiation for 10 min in different conditions (no laser, 808-nm laser, 660-nm laser, 808/660-nm laser). In parallel, standard CCK-8 assay was also conducted to assess the cell viability after photothermal–photodynamic treatment.

### Cancer Cell Uptake Behaviour and *in Vitro* MR Imaging Studies of MCPM NPs

MDA-MB-231 cells (8 × 10^4^ cell/well) were seeded on the coverslips in a six-well plate overnight. Thereafter, MCPM NPs were added to each well at a concentration of 0.3 mg ml^−1^ and incubated for another 4 h. Next, cells were rinsed, fixed, and stained with DAPI, followed by observation with CLSM (LS880, Zeiss, Oberkochen, Germany). With respect to *in vitro* MR imaging, MDA-MB-231 cells (1 × 10^5^ cell/well) were seeded on a six-well culture dish for 24 h. Thereafter, MCPM NPs at different concentrations (0, 0.03, 0.1, 0.3 mg ml^−1^) were added in each well. Then, the cells were rinsed, trypsinized, centrifuged, and transferred into Eppendorf tubes for MRI. The *in vitro* MR images were obtained with the MR system and parameters described previously.

### Detection of *in Vitro* Reaction Oxygen Species

MDA-MB-231 cells (1 × 10^5^ cell/well) were seeded on the coverslips in a six-well plate and kept overnight for cell attachment. Thereafter, MCPM NPs were added in each well at a concentration of 0.3 mg ml^−1^ and underwent different laser irradiations for 10 min (no laser, 808-nm laser, 660-nm laser, 808/660-nm laser). Thereafter, cells were rinsed thrice in PBS, fixed with 4% paraformaldehyde solution, and mounted with mounting medium containing 2,7-dichlorodihydrofluorescein diacetate (DCFH-DA) and 4′,6-diamidino-2-phenylindole (DAPI), respectively. Subsequently, the samples were evaluated under a CLSM (LS880, Zeiss, Germany).

### Live/Dead Cell Staining Assay

Calcein-AM (green fluorescence) and propidium iodide (PI, red fluorescence) were employed to assess cell viability before and after laser irradiation (808 nm, 1.0 W cm^−2^, 10 min; 660 nm, 100 mW cm^−2^, 10 min), with identical manipulations to the CCK-8 assay. With the addition of Calcein-AM and PI into each well, cells were incubated for 30 min without CCK-8 treatment. Thereafter, the cells were observed under CLSM for photography.

### *In Vivo* Biodistribution, Metabolism, MRI, and Fluorescence Imaging

The animal experiments were performed in accordance with the protocol approved by the Institutional Animal Care and Use Committee of Xuzhou Medical University (ethical approval number: 202007W001). For the establishment of the mouse tumor model, MDA-MB-231 cells (2 × 10^6^ cells in 150 μl) were subcutaneously injected in the right axilla in BALB/C nude mice (female, 6 weeks, 15–17 g).

For the biodistribution study, BALB/C nude mice were intravenously injected with MCPM NPs (10 mg ml^−1^, 0.2 ml). The blood was collected *via* the caudal vein 40 μl/time at time points of 0.5, 1, 2, 3, 4, 6, 8, 12, and 24 h post-injection. The blood samples were digested with 40 μl nitric acid (analytically pure) and then heated in an 80°C water bath for 30 min and diluted to 5 ml by addition of ultrapure water. The Mn content in these samples was determined with inductive coupled plasma mass spectrometry (ICP-OES).

The mouse tumor models were deployed in *in vivo* MR imaging in the case of the tumor size of 5 mm, wherein MCPM NPs (10 mg kg^−1^) were systematically administered into the mouse tumor models *via* the caudal vein. Thereafter, murine MR imaging was performed at different time points pre- and post-administration (0.25, 0.75, 1, 2, 3, 4, 6, 8, 12, and 24 h). For *in vivo* fluorescent imaging, MCPM NPs (10 mg kg^−1^) were injected into the mice. The images were obtained on a fluorescence imaging system (Bio-Rad, Hercules, CA, USA) at different time points pre- and post-administration (0.25, 0.75, 1, 2, 3, 4, 6, 8, 12, and 24 h).

### *In Vivo* Anticancer Effect

The *in vivo* antitumor efficacy of PTT–PDT combination therapy was investigated in MDA-MB-231 nude mice (with tumor volume ∼100 mm^3^). The mice were randomized into six groups (three mice/each) and received an injection of 200 μl saline, saline + 808/660 nm laser, MCPM NPs, MCPM NPs + 808 nm laser, MCPM NPs + 660 nm laser, and MCPM NPs + 808/660 nm laser (0.3 mg ml^−1^), respectively. At 2 h post-injection, the tumor areas of mice were conducted to laser irradiation for 10 min (808 nm, 1.0 W cm^−2^; 660 nm, 100 mW cm^−2^, 10 min). The temperature was recorded by a FLIR thermal camera. The body weight and tumor volume in the mice were determined every other day [tumor volume= (tumor length tumor width)^2^/2]. All the treatments were repeated every 3 days from day 1 to day 14.

### *In Vivo* Biosafety Evaluation

The *in vivo* biosafety of the MCPM NPs was assessed in BALB/C mice with the biochemistry indexes and histology analysis. Healthy BALB/C mice were randomized into control group and three experimental groups. Mice in the experiment group received an intravenous injection of aqueous MCPM NPs (10 mg kg^−1^), with blood samples collected for blood biochemical analyses at 1, 7, and 21 days post-injection. The major organs such as the heart, liver, spleen, lungs and kidneys of the mice were isolated for histological analysis with hematoxylin and eosin (H and E) staining, followed by histological analyses under a microscope.

### Statistical Analysis

All data in this experiment are expressed as mean ± SD. One-way ANOVA was employed for comparison between multiple samples. Student’s *t*-test was adopted to evaluate the between-group statistical significance, with *p* < 0.05 considered as statistically significant in all cases.

## Results and Discussion

### Characterization of the Synthesized Nanoparticles

**FIGURE 1:**
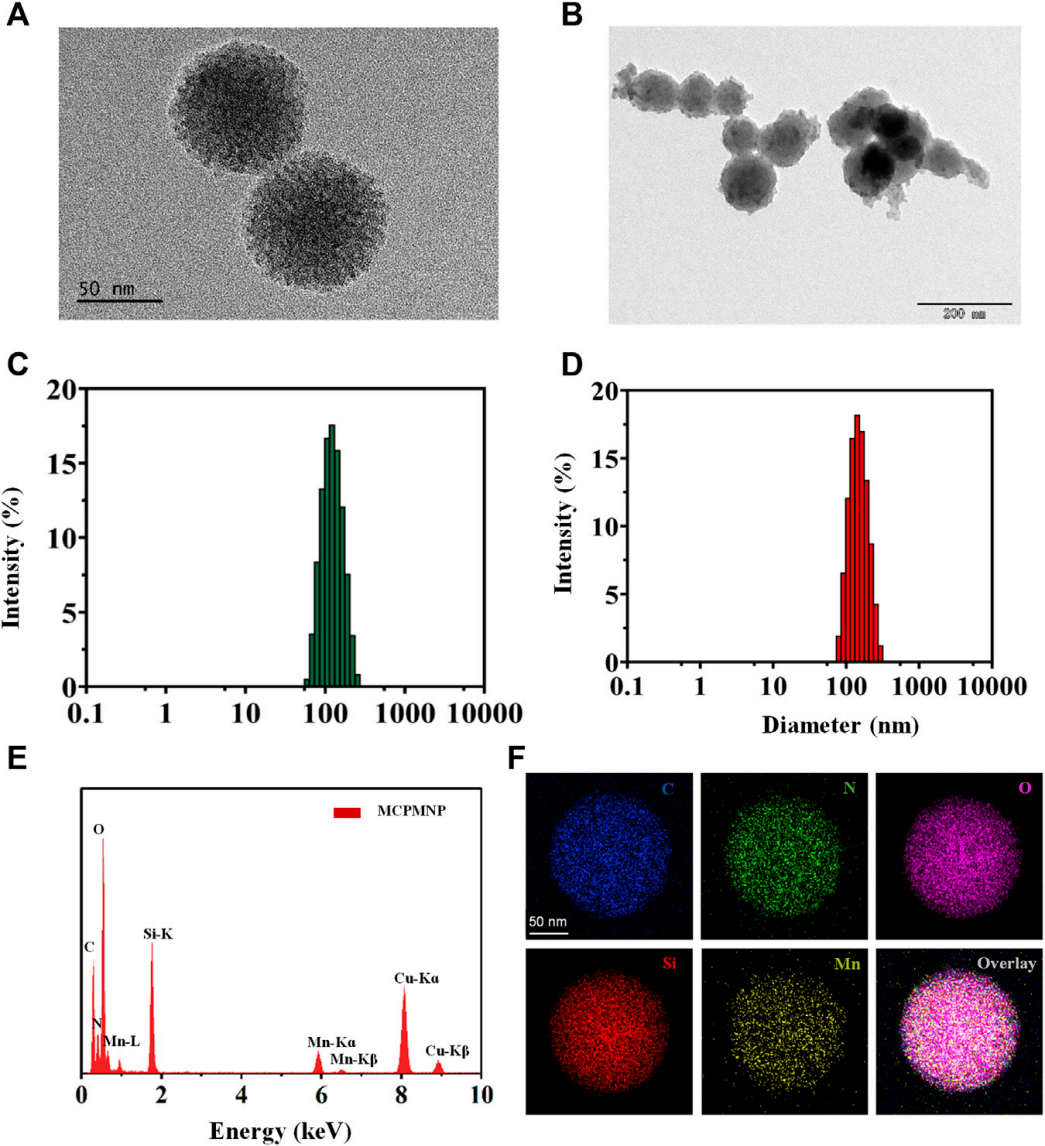
Characterization of MSN and MCPM NPs. **(A)** TEM images of MSN. **(B)** TEM images of MCPM NPs. **(C)** Hydrodynamic size distribution of MSN. **(D)** Hydrodynamic size distribution of MCPM NPs. **(E)** EDS spectrum of MCPM NPs. **(F)** The elemental mapping images of MCPM NPs.

The strategy for the preparation of MCPM NPs is illustrated in Scheme 1. The TEM images of as-synthesized MSN in Figure 1A demonstrated that the MSN was almost uniform in size with a spherical morphology. The estimated mean diameter was 125 ± 2.46 nm (Figure 1C). As for the MCPM NPs, there were evident changes in morphology, with MCPM NPs presenting a near-spherical appearance and relatively smooth surface (Figure 1B), with an estimated average size of 139 ± 1.70 nm (Figure 1D).

For T_1_-weighted MR imaging, Mn ions were chelated inside the structure of PDA by dispersion of MSN-Ce6@PDA in a solution of Tris and manganese (II) chloride tetrahydrate. To explore the element composition of MSN-Ce6@PDA (Mn), all the major elements such as C, N, O, Si, and Mn were detected within the EDS spectrum (Figure 1E) and the elemental mapping images (Figure 1F).

**FIGURE 2:**
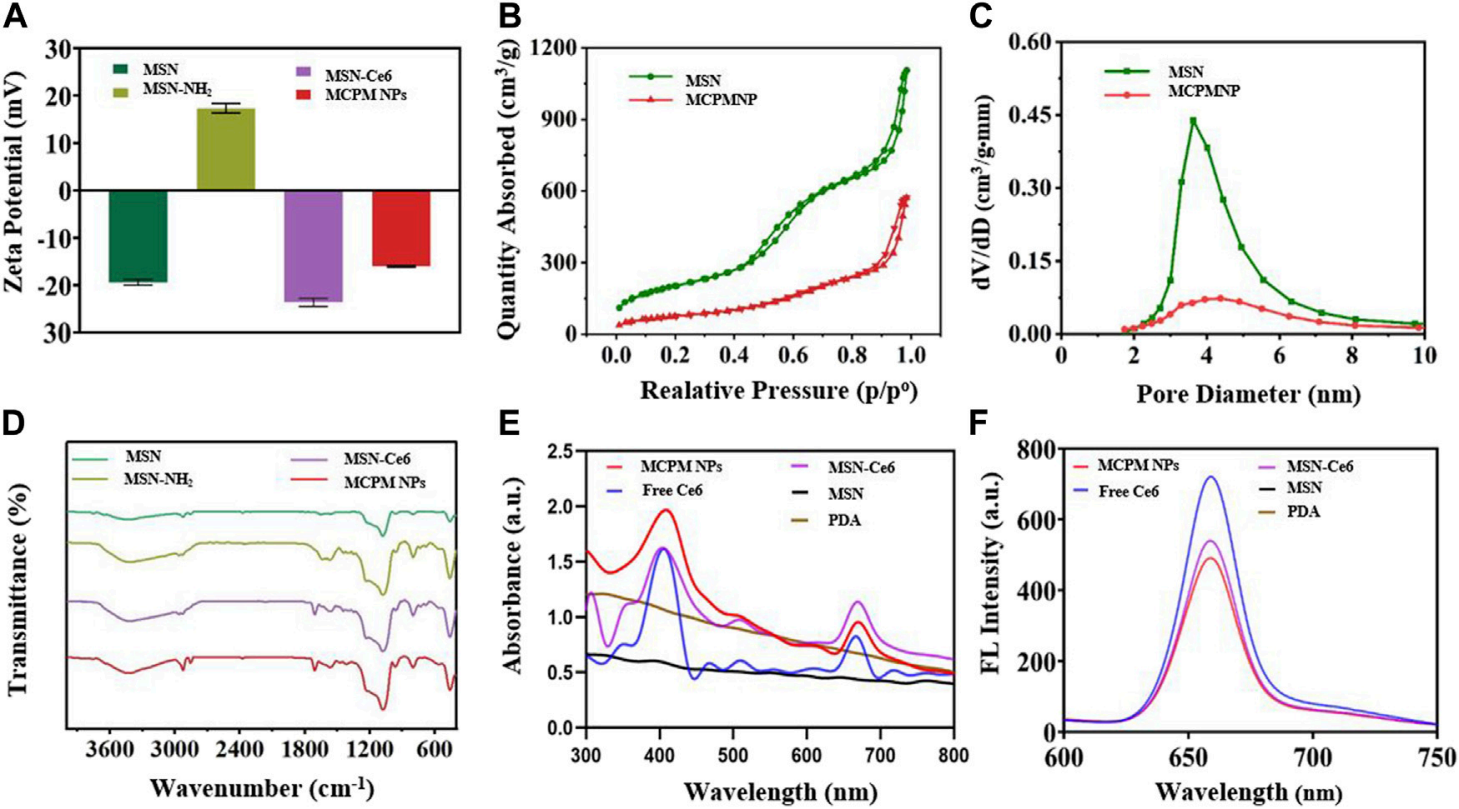
Zeta potentials and spectra of different nanoparticles. **(A)** Zeta potentials. **(B)** Nitrogen adsorption–desorption isotherms of MSN and MCPM NPs. **(C)** Pore size distribution of MSN and MCPM NPs. (The green line represents the MSN, and the red line represents the MCPM NP). **(D)** FT-IR spectra. **(E)** UV-Vis absorption spectra. **(F)** Fluorescence spectra.

A series of zeta potential measurement is illustrated in Figure 2A. The zeta potential of MSN and MSN-NH_2_ was measured at -19.4 ± 0.51 mV and 17.3 ± 0.81 mV attributed to the hydroxyl groups and amino groups, respectively. After conjugation of Ce6, the zeta potential decreased to −23.63 ± 0.73 mV because the Ce6 molecule contains three negatively charged carboxyl groups. After PDA coating and manganese ion coordination, the zeta potential of MCPM NPs increased. In contrast, PDA contains a large amount of amine phenolic hydroxyl (anionic) groups with negative charges, whereas a small quantity of Mn contains positive charges. Thus, after the coordination of Mn^2+^, the MCPM NPs gain a negative surface charge of −16.03 ± 0.12 mV. The change in a series of zeta potential indicated the successful preparation of MCPM NPs. The MSN-Ce6@PDA and MCPM NPs exhibited good dispersity and stability in PBS even for 21 days, whereas MSN-Ce6 and free Ce6 displayed unsatisfactory stability (Supplementary Figures S1, S2). Ce6 has a hydrophobic potency, hence the high dissolution in DMSO rather than PBS. MSN-Ce6 showed good stability in the initial 2 days but precipitated after 7 days (Supplementary Figure S1). Besides, the hydrate particle sizes of MCPM NPs show little change at the 4°C storage condition (Supplementary Figure S3). The laser irradiation treatment also exhibited small change to the hydrodynamic size of MCPM NPs (Supplementary Figure S4). In addition, the MCPM NPs also possess good stability, which enable good T_1_-weighted MR stability in PBS for a long term (Supplementary Figure S4).

As illustrated in Figures 2B,C, N_2_ sorption isotherms for MSN and MCPM NPs were determined to explore the effect of the Ce6 conjugating and PDA coating on the surface area and pore volume of the MSN. The BET and BJH analyses yielded a highly specific surface area of 728.4 cm^2^g^−1^ and a mean pore size of 10.5636 nm for the MSNs. After a series of synthetic procedures, the decreased BET surface area (275.4 cm^2^g^−1^) and mean pore size (7.4 nm) of the prepared MCPM NPs confirmed the successful assembly. In addition, the reduced volume of absorption and the diminished pore size manifested the decrease of mesoporous silica/nanoparticle composites, further validating the successful coating of PDA.

To further verify the successful assembly of MSN, a comparison of MSN, MSN-NH_2_, MSN-Ce6, and MCPM NPs was performed by the FTIR spectrum of the different formulations. As depicted in Figure 2D, absorbance peaked at approximately 1,076 cm^−1^ of the MSN, MSN-NH_2_, MSN-Ce6, and MCPM NP samples, which could be the stretching of Si–O–Si groups, verifying the successful synthesis of MSN. Compared with the unmodified MSN, the aminated mesoporous silica showed a new peak around 1,556 cm^−1^, which was related to N–H bending of the amino group, indicating the successful amination. The new absorbance peak appeared at 1,710 cm^−1^ for MSN-Ce6, and the merged band was formed consequent to the C = O stretching in the amide bond, indicative of the success of the amide reaction. Furthermore, the presence of PDA on the surface of MSN-Ce6 could be validated *via* the onset of absorbance peaks at 1,619 cm^−1^ designated to the superposition of N–H bending and C–C stretching of aromatic components, 1,485 cm^−1^ (N–H scissoring), and 1,282 cm^−1^ in response to C–O stretching of the phenol of PDA (Hu et al., 2016; Sun et al., 2018). Given that, the MCPM NPs were successfully synthesized.

No absorbance peaks were evidenced in the controlled PDA and MSN groups, with typical UV-vis peaks of free Ce6 at 400 and 660 nm, and the fluorescent emission peak of free Ce6 at 660 nm. Despite the presence of an evident red shift in the UV-vis peak and fluorescence emission peak of MSN-Ce6 and MCPM NPs at 667 nm compared to Ce6, Ce6 was successfully conjugated to MSN (Figure 2E).

The fluorescent efficiency of Ce6 is pivotal to PDT efficacy. As delineated in Figure 2F, compared with the potent fluorescence of free Ce6, the peak in MSN-Ce6 and MCPM NPs was at approximately 660 nm, which is slightly affected owing to the π–π stacking of molecules. We speculated that the MSN-Ce6 and MCPM NPs could still be suitable for PDT.

### Photothermal and Singlet Oxygen Generation Performance

**FIGURE 3:**
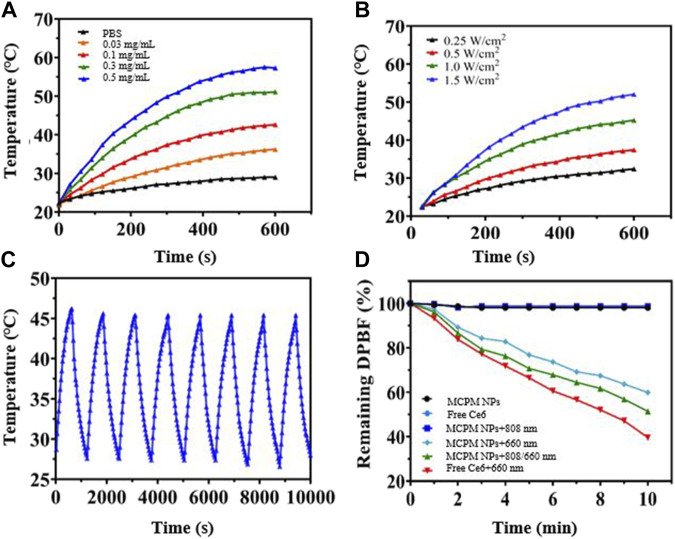
Photothermal and singlet oxygen generation performance of MCPM NPs. **(A)** Temperature curves at different concentrations of MCPM NPs irradiated under 1.0 W cm^−2^. **(B)** Temperature curves of MCPM NP solution under 808-nm laser irradiation with different powers. **(C)** Heating and cooling curves of MCPM NPs irradiated by a 1.0-W/cm^2^ 808 nm laser over eight ON/OFF cycles. **(D)** Singlet oxygen generation of nanoparticles under laser irradiation.

Given the successful PDA coating on the MSN, we investigated the photothermal performance of the MCPM NP dispersion under the irradiation of the 808-nm laser. The hydrodynamic size of MCPM NPs with or without laser irradiation exhibited little difference (Supplementary Figure S3). As depicted in Figure 3A; Supplementary Figure S5, MCPM NPs were rapidly heated to a threshold in a concentration-dependent manner. At the end of a 10-min exposure to the 808-nm laser (1.0 W cm^−2^), the MCPM NP dispersion of 0.03–0.5 mg ml^−1^ was quickly heated to 36.2°C–57.3°C from ambient temperature. In contrast, the temperature of the PBS solution was only heated to 29.0°C. Moreover, under irradiation of the 808-nm laser at different intensities (0.25–1.5 W cm^−2^), the MCPM NP solution (0.3 mg ml^−1^) also presented an evident temperature rise from 32.4°C to 52°C with an obvious NIR power-dependent pattern (Figure 3B), indicating the superior conversion capacity from 808-nm laser energy to thermal energy. In parallel, the photothermal stability was also evaluated *via* repeated laser irradiation to MCMP NP dispersion for eight on/off cycles. In Figure 3C, the dispersion was steadily heated to the peak temperature in the eight heating/cooling cycles, authenticating its excellent photothermal stability (Figure 3D). Thus, the efficient thermal treatment of the PDA shell validated the stability and continuum of the thermal gradient for the thermophoretic propulsion of the MCPM NPs.

The DPBF kit was employed to evaluate the generation of singlet reaction oxygen species (ROS). DPBF was remarkably degraded by the ROS, with the degree of degradation closely dependent on the ROS generated by MCPM NPs. The 808/660-nm laser group generated the most ROS. This result indicated that the 808-nm laser triggered the release of Ce6, contributing to the greater ROS generation performance than MCPM NPs under the 660-nm laser alone. A previous report also revealed that the degradation of the mesoporous silica nanocarrier could facilitate drug release (Shao et al., 2020). Furthermore, some other studies also indicated that the MSNs with a large mesopore of ∼10 nm revealed a much faster release (Shen et al., 2014). As mentioned in Figure 2C, our MSNs possessed a mean pore size of 10.5636 nm; therefore, the matrix degradation of MSN probably achieved a fast Ce6 release, which probably induced more ROS generation.

### *In Vitro* Biocompatibility, Cancer Cell Uptake Behaviour and *in Vitro* MR Imaging Studies of MCPM NPs

#### *In Vitro* Biocompatibility

**FIGURE 4:**
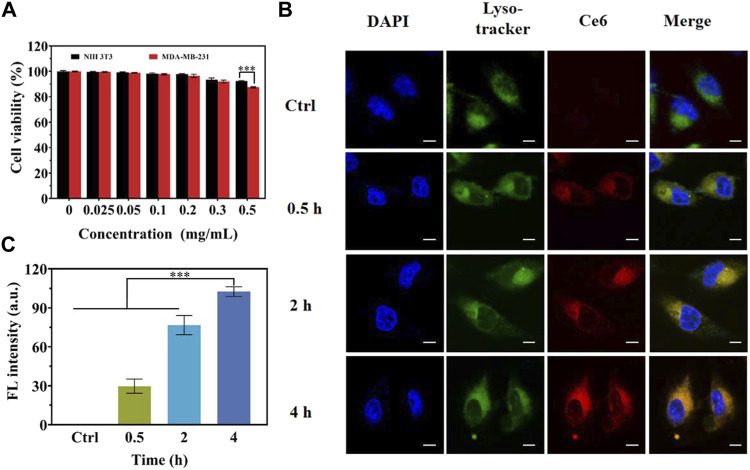
*In vitro* cytotoxicity and cellular uptake behavior of MCPM NPs. **(A)** *In vitro* cytotoxicity assay of MCPM NPs in NIH3T3 and MDA-MB-231 cells. **(B)** CLSM images of MDA-MB-231 cells with MCPM NP treatment for different incubation durations. **(C)** Profiles of respective fluorescence intensities for the MDA-MB-231 cells in **(B)**. ***p < 0.001.

The biocompatibility, which is one of the critical evaluation criteria for the selection of nanotheranostic agents. *In vitro* CCK-8 assay was used to investigate the potential cytotoxicity of as-prepared samples. As delineated in Figure 4A, no evident toxicity to the two cell lines was explored at all the concentrations involved of MCPM NPs after 24 h incubation by the CCK-8 assay.

#### Cancer Cell Uptake Behavior

 Cellular internalization efficiency of MCPM NPs is vital to cellular imaging and cancer treatment. To further explore the intracellular distribution, MDA-MB-231 cells under co-incubation with MCPM NPs within different durations as well as observation under CLSM. Indeed, most of the MCPM NPs exhibited red fluorescence and were distributed in the lysosome of MDA-MB-231 cells after 1 h of incubation. With the prolonged incubation, an enhanced cellular uptake in MDA-MB-231 cells was evidenced by the brighter red fluorescence (Figures 4B,C).

#### *In Vitro* MRI

**FIGURE 5:**
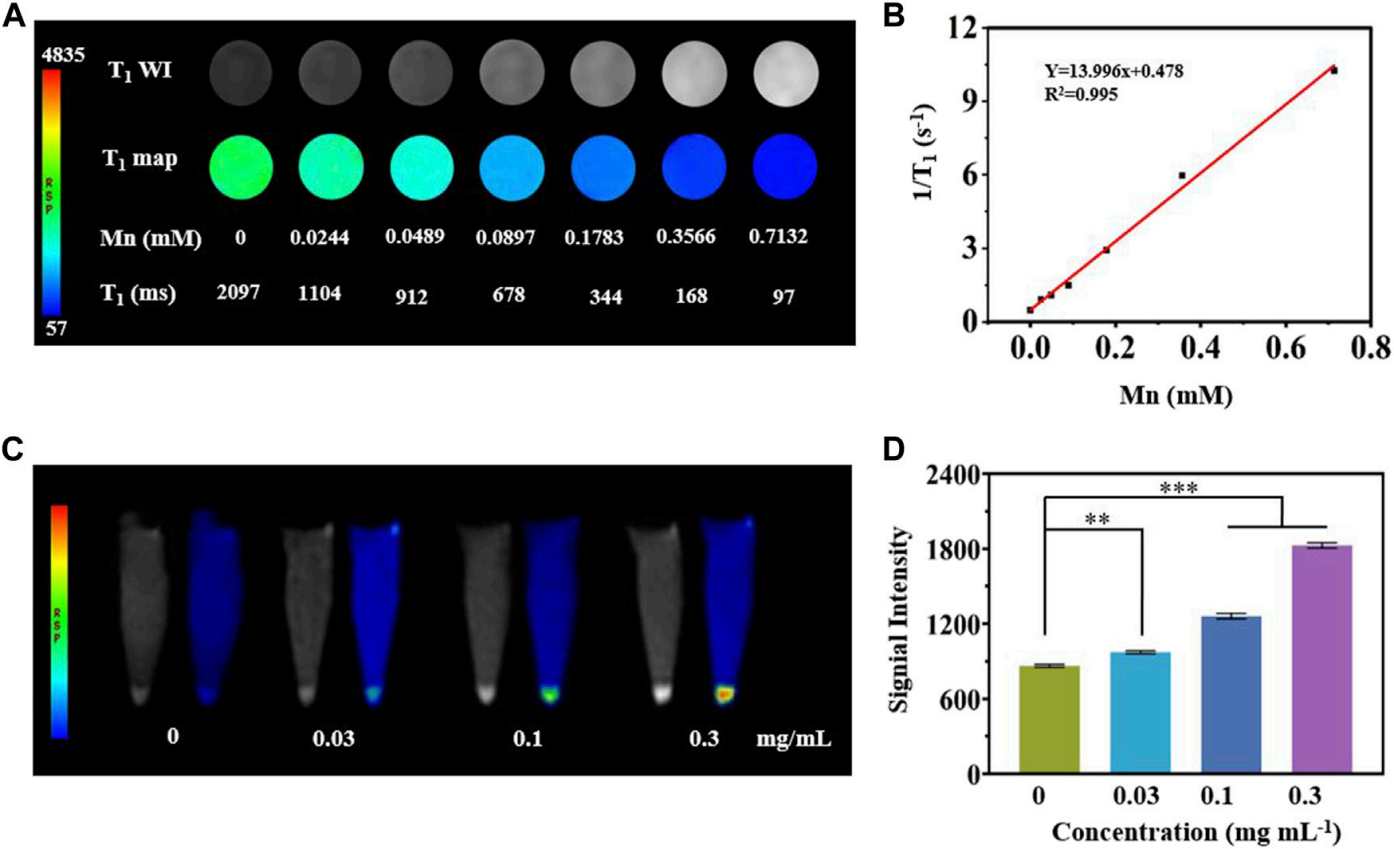
*In vitro* MRI studies of MCPM NPs. **(A)** T_1_-weighted phantom images and pseudo-color images of MCPM NPs at different Mn concentrations. **(B)** The longitudinal relaxation rate of MCPM NPs serves as an index for a series of Mn concentrations. **(C)** T_1_-weighted MRI images of MCPM NPs for MDA-MB-231 cells at a cascade of Mn concentrations. **(D)** Profiles of T_1_ MRI signals of the MDA-MB-231 cells in **(C)**. **p < 0.01, ***p < 0.001.

A T_1_-weighted MR assessment was conducted for the feasibility of MCPM NPs as theranostic nanoprobes. The brightness of phantom images was consistent with the gradation of Mn concentrations (Figure 5A), indicating the linear dependence of MR signals on Mn concentrations. The r_1_ relaxivity value of MCPM NPs was 13.996 mM^−1^S^−1^ (Figure 5B), 3.20-fold that of commercial product Gd-DTPA (4.37 mM^−1^S^−1^). The higher r1 value of MCPM NPs suggested that the presence of a PDA coating facilitated the absorption of more Mn^2+^ molecules, thus generating a stronger magnetic resonance signal.

As revealed in Figure 5C, MDA-MB-231 cells treated by MCPM NPs exhibited a brighter signal compared to the group without MCPM NP treatment, verifying the superiority of the MCPM NPs in MRI of tumor cells. Moreover, in the MCPM NP groups, the brightness of the signal was concentration-dependent (Figure 5D). These results demonstrated that MCPM NPs could be accumulated inside MDA-MB-231 cells, thereby enhancing the MRI outcome.

### *In Vitro* Generation of Reaction Oxygen Species and Antitumor Efficacy

#### *In Vitro* Generation of Reaction Oxygen Species

**FIGURE 6:**
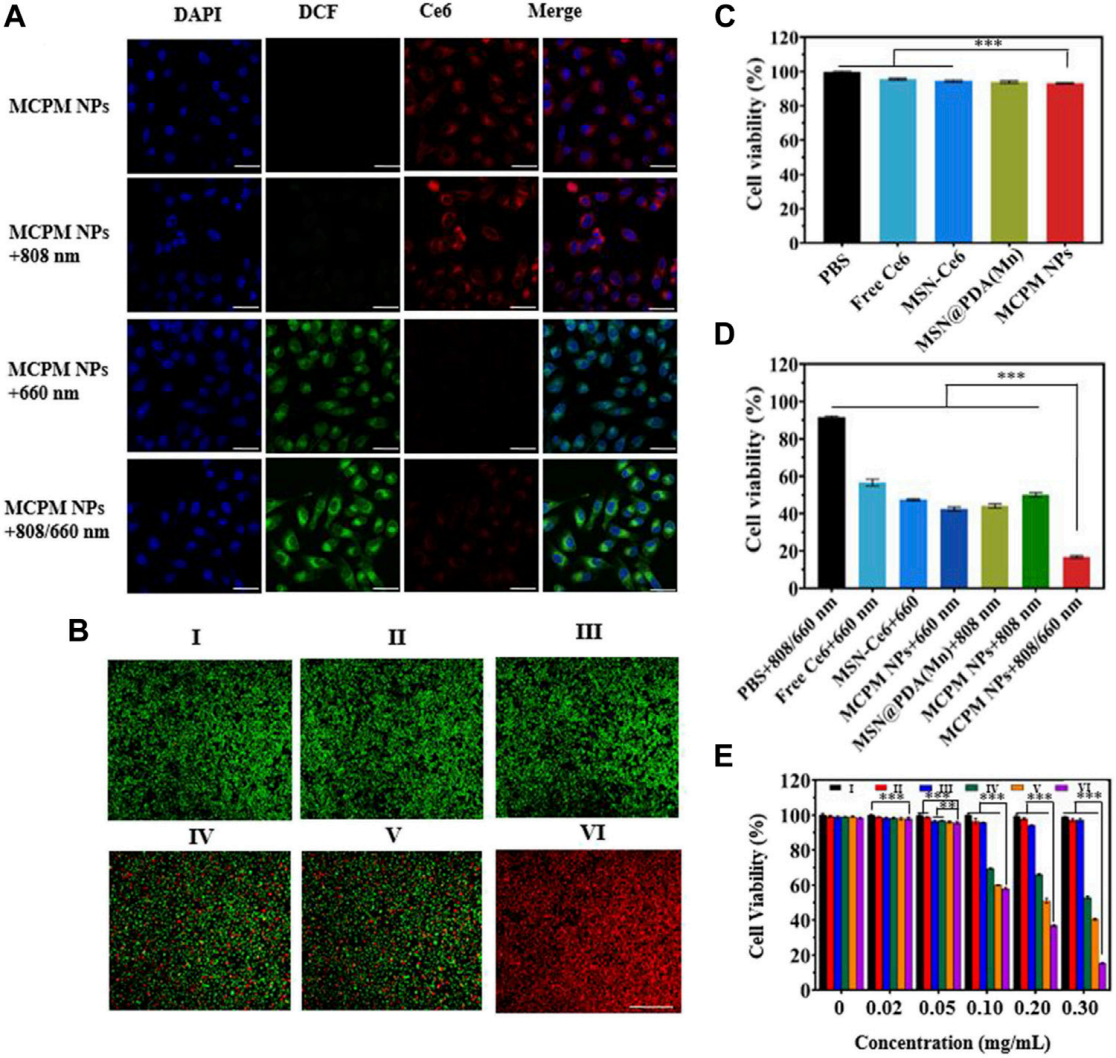
*In vitro* performance of MCPM NPs tested in MDA-MB-231 cells. **(A)** Detection of singlet oxygen in MDA-MB-231 cells undergoing treatment with MCPM NPs under various modes of laser irradiation with DCFH-DA assay, scale bar = 20 μm. **(B)** Staining profiles of viable and dead MDA-MB-231 cells treated with MCPM NP-based PTT/PDT. **(C)** Viability of MDA-MB-231 cells treated with NPs. **(D)** Viability of MDA-MB-231 cells treated with NPs and laser irradiation. **(E)** Cell viability at different concentrations of NP-based PTT/PDT. The irradiations are conducted at 808 nm (1.0 W cm^−2^, 10 min) and 660 nm (100 mW cm^−2^, 10 min) for the above experiments. Data are shown as mean ± S.D. (*n* = 6), **p < 0.01, ***p < 0.001. (Groups I-VI are designated as PBS, MCPM NPs, PBS + 808/660 nm laser, MCPM NPs + 808 nm laser, MCPM NPs + 660 nm laser, and MCPM NPs + 808/660 nm laser groups, respectively).

To explore the PDT effect of MCPM NPs, the detection of intercellular ROS generation was performed with intracellular ROS indicator DCFH-DA as a probe. The presence of ROS could oxidize DCFH-DA (with no fluorescence) into DCF (with green fluorescence). Moreover, the green fluorescence intensity was positively correlated with the ROS production. As illustrated in Figure 6A, bright green fluorescence was evident in the presence of MCPM NPs with the 660-nm laser or 808/660-nm laser. In addition, the latter group exhibited even stronger green fluorescence than the 660-nm laser-irradiated group, whereas no fluorescence was visible in the group of MCPM NPs with the 808-nm laser. These results demonstrated the effective synergism of MCPM NPs in the ROS generation for cancer treatment.

#### *In Vitro* Antitumor Efficacy

Red fluorescence is evident in dead cells rather than viable cells, by which observation of the fluorescence profiles could serve to reflect the killing effect of different treatments. As shown in Figure 6B, red fluorescence was negligible in the control group (PBS, I), MCPM NP group (II), and PBS + 808/660-nm laser group (III) without any nanocomposites. Nevertheless, part of the red fluorescence appeared in the presence of MCPM NPs with the 808-nm (IV) or 660-nm laser (V) during cell incubation. Notably, a full-field view of red fluorescence was observed after treatment of MCPM NPs coupled with the dual-wavelength 808/660-nm laser (VI).

CCK-8 assay was conducted to investigate the cytotoxicity of various samples to MDA-MB-231 cells under different regimens. The cell viabilities after treatment with free Ce6, MSN-Ce6, MSN-Ce6, MSN@PDA (Mn), and MCPM NPs were kept above 90% after 24 h of incubation with dark treatment (Figure 6C). Furthermore, the cell viabilities were reduced after laser treatment, with the low point in the MCPM NPs plus combined treatment group (Figure 6D). Moreover, the killing effect of the IV group exhibited a concentration-dependent manner (Figure 6E).

### *In Vivo* Biodistribution, Metabolism, MR, and Fluorescence Imaging

**FIGURE 7:**
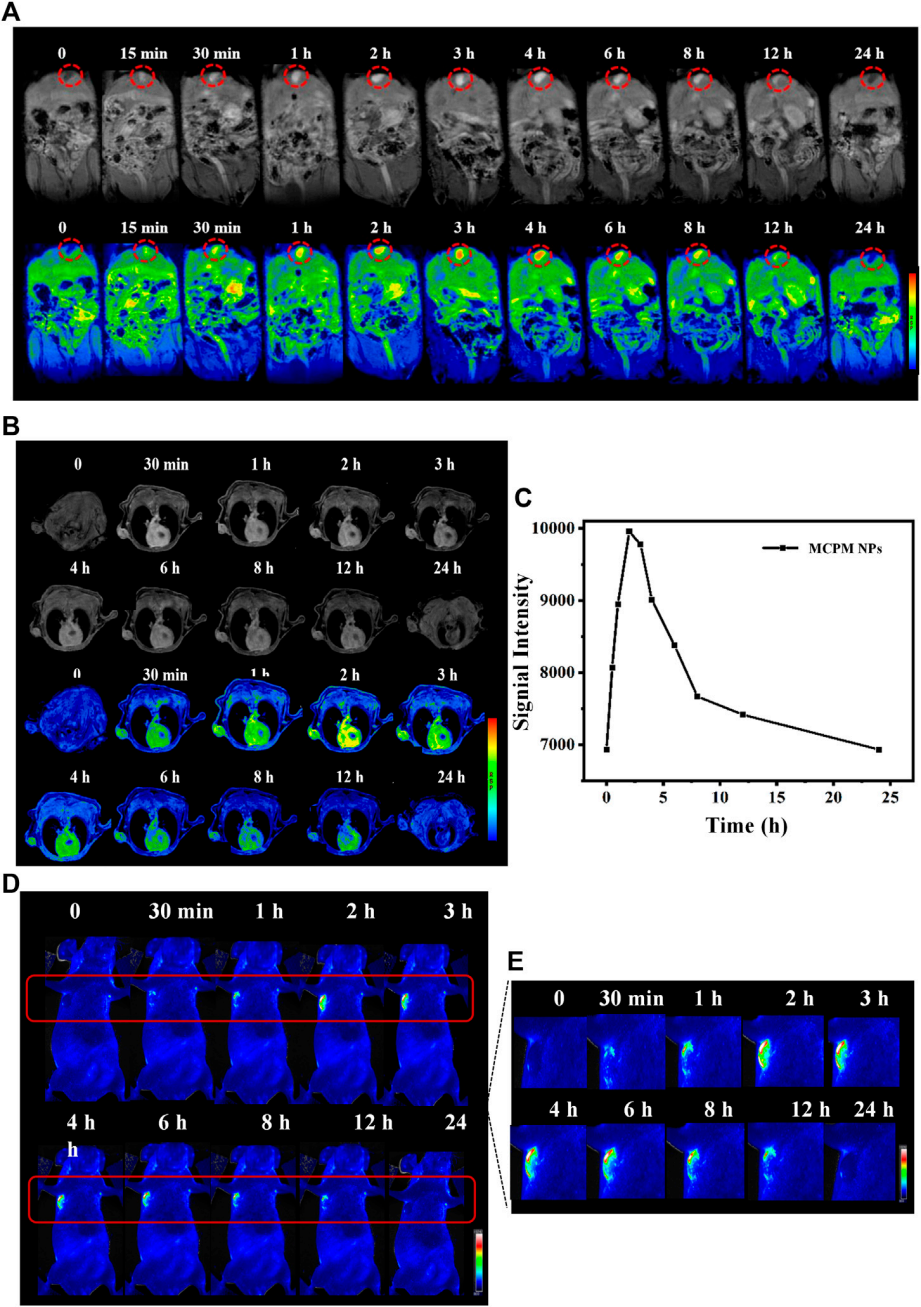
MRI/FL ability of MCPM NPs. **(A)** *In vivo* T_1_-weighted MR images and pseudo-color images of mice with subcutaneous MDA-MB-231 tumor after i.v. injection of MCPM NPs at different time points. Inside red circle is the gallbladder. **(B)** The corresponding changes in single intensities in the tumor areas. **(C)** *In vivo* FL images of mice with subcutaneous MDA-MB-231 tumor after i.v. injection of MCPM NPs at different time points. **(D)** The regions in red boxes in **(C)** were amplified and magnified in the right panel.

Given the successful T_1_-weighted MRI of MDA-MB-231 cells by MCPM NPs, we further investigated their possible application for *in vivo* imaging under a 3.0-T MRI system. The MCPM NPs were intravenously administered into the BALB/c nude mice with heterografted breast cancer. Then, mice were performed with MRI at various time points pre- and post-injection. Prior to injection of MCPM NPs, an MR signal of relatively low intensity was identified across the mice including the tumor region. Nevertheless, following intravenous administration of MCPM NPs, the MR signal was markedly enhanced in comparison to the pre-injection state. A significantly enhanced MR signal was observed in the liver and gallbladder within 4 h post-injection of MCPM NPs, which was attenuated afterward, implying the presence of a possible clearance route of the nanotheranostic agent presumably *via* hepatobiliary excretion (Figure 7A). Moreover, the gradual brightness in the tumor region was consistent with the prolonged duration after injection, with the onset of the peak at 2–3 h post-injection and decline in an MR signal intensity within 24 h (Figure 7B). Furthermore, we quantified the signal value of the tumor region and identified the presence of enhanced T_1_-weighted MR signals after the administration of MCPM NPs (Figure 7C).

A similar approach was utilized by *in vivo* fluorescence imaging. Accordingly, no contrast enhancement was observed in tumors before i.v. injection of the MCPM NPs. The presence of sensitive brightness in the tumor region appeared at 30 min, with a strong contrast enhancement at 2–3 h, following signal reduction within 24 h (Figures 7C,D). These results confirmed the accumulation and retention of MCPM NPs in tumors. Therefore, the MCPM NPs can be effectively used in MDA-MB-231 nude mice.

### *In Vivo* PTT-PDT Performance

**FIGURE 8:**
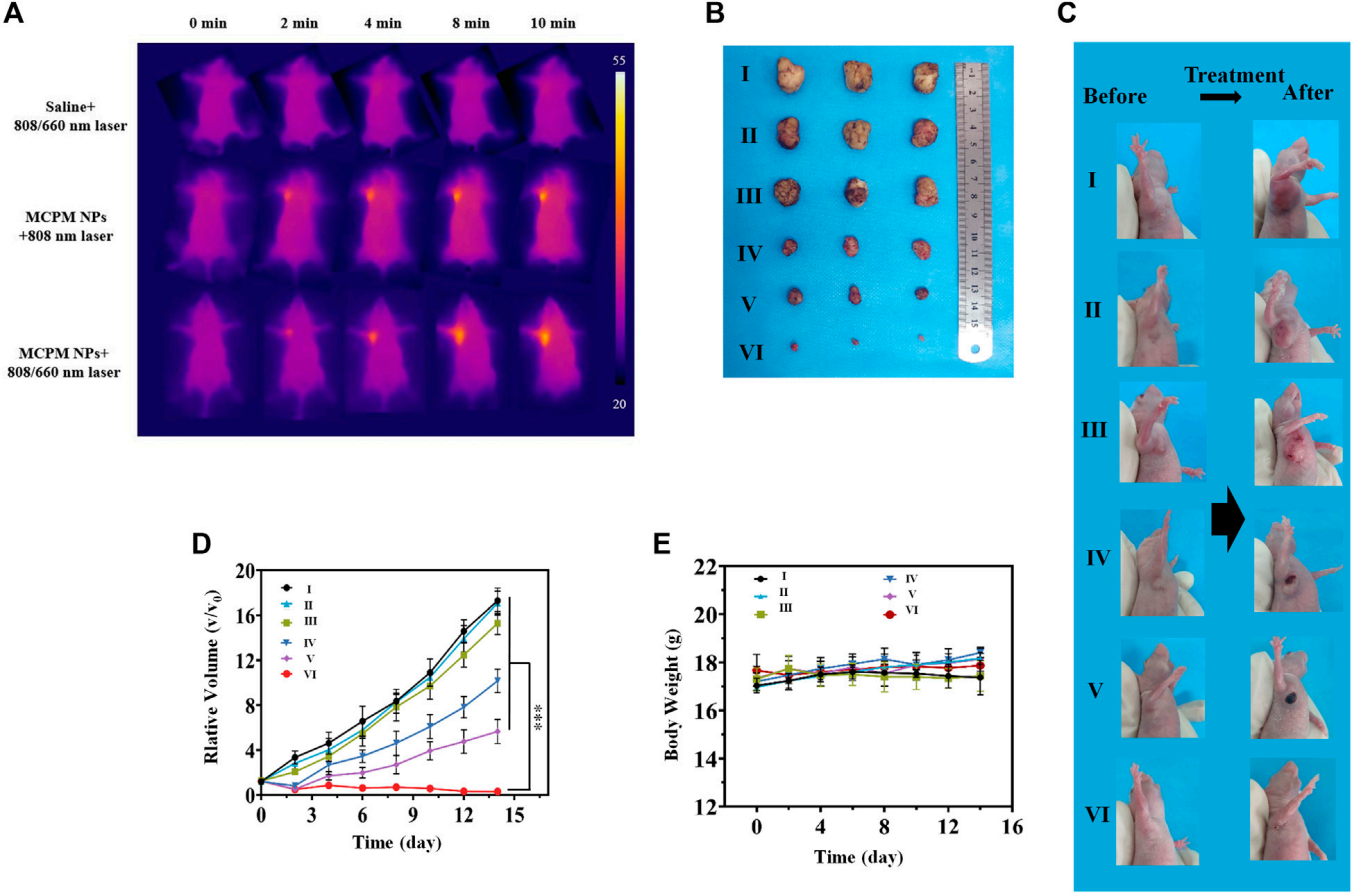
*In vivo* PTT-PDT performance. **(A)** Thermal images of the MDA-MB-231 tumor model mice upon different treatments. **(B)** Photographs of tumors from different treatment groups. Average tumor weight after different treatment. **(C)** Photographs of mice with subcutaneous MDA-MB-231 tumor following different regimens. **(D)** Change curves of relative tumor sizes. ***p < 0.001. **(E)** Change curves of body weight of mice with MDA-MB-231 tumor following different regimens. Data are shown as mean ± SD, (*n* = 3), (Groups I-VI are designated as saline, MCPM NPs, saline + 808/660 nm laser, MCPM NPs + 808 nm laser, MCPM NPs + 660 nm laser, and MCPM NPs + 808/660 nm laser groups, respectively).

Considering the *in vivo* MRI and fluorescence imaging outcome, the *in vivo* cancer therapy with MCPM NPs was then evaluated (Figure 7). The temperature of the tumor area rapidly rose to a plateau of 46.5°C and then remained stable (Figure 8A) under laser irradiation. In contrast, the temperature of the saline group barely changed. When the tumor volume grew to 100 mm^3^, the mice were randomly divided into six groups: (I) saline, (II) MCPM NPs, (III) s plus 808/660 nm laser, (IV) MCPM NPs plus 808 nm laser, (V) MCPM NPs plus 660 nm laser, and (VI) MCPM NPs plus 808/660 nm laser (808 nm laser, 1.0 W cm^−2^, 10 min; 660 nm, 100 mW cm^−2^, 10 min). Saline and MCPM NPs were injected into each mouse *via* the tail vein at 10 mg kg^−1^ for MCPM NPs. Then, 2 h later, those mice were performed with different treatments as described previously. After laser irradiation, the tumor volumes were measured by a vernier caliper every other day for 2 weeks. In the photothermal–photodynamic combination therapy group, the MCPM NPs plus 808/660-nm laser yielded a significant inhibition of tumor growth (Figures 8B,C). MCPM NPs plus laser irradiation (IV, V, and VI), for the initial several days, exerted a slight inhibitory effect on the tumor growth, which however resumed thereafter. No significant effect on tumor growth was observed in the control (I) and the remaining two treatment groups (II, III) (Figure 8D), while the body weight barely changed (Figure 8E).

### Biochemistry Index and Histological Analysis

**FIGURE 9:**
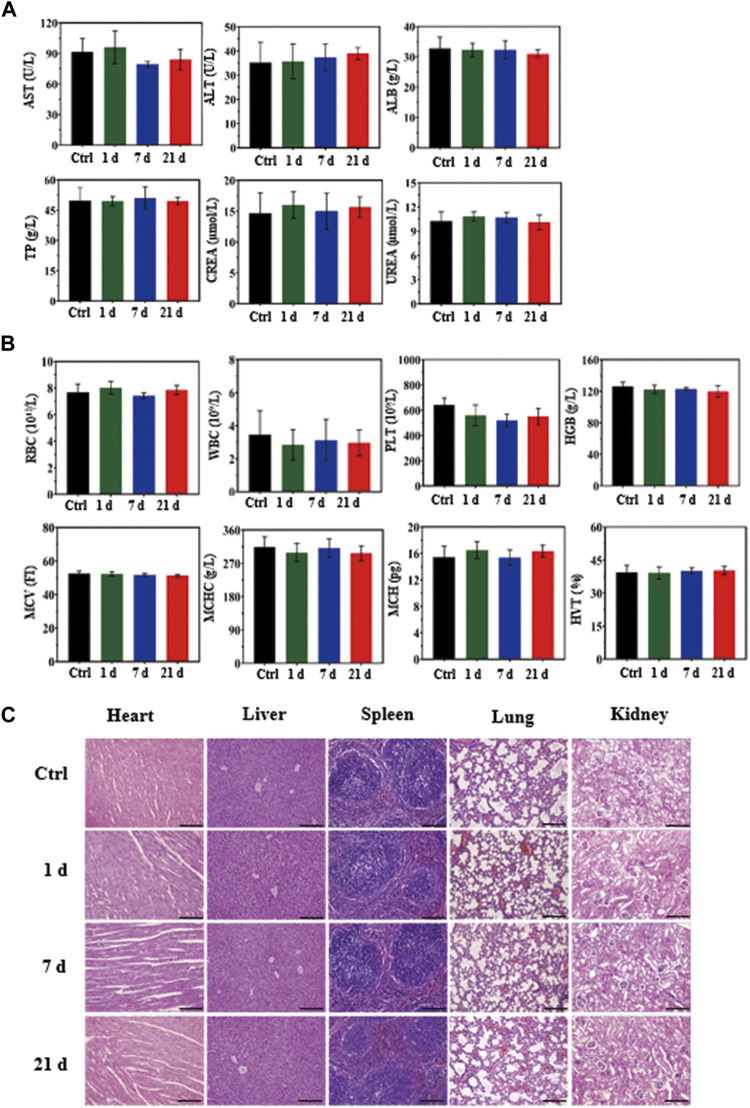
*In vivo* biosafety assay of MCPM NPs (10 mg/kg). **(A)** Biochemical analysis of mouse serum before (control, day 0) and after injection of MCPM NPs for days 1, 7, and 21. **(B)** Histological images of the heart, liver, spleen, lungs, and kidneys from healthy mice injected with MCPM NPs at days 1, 7, and 21, and control mice. The visceral sections underwent staining with hematoxylin and eosin (H and E) and observation under an optical microscope, scare bar = 100 μm.

The biological safety of MCPM NPs *in vivo* was assessed in healthy mice by means of blood biochemistry and histological analysis of organs at days 1, 7, and 21 after injection. No marked differences were observed with respect to the levels of indicators including blood aspartate aminotransferase, alanine aminotransferase, albumin, total protein, creatinine, urea, erythrocytes, leukocytes, platelets, hemoglobin, mean corpuscular volume, mean corpuscular hemoglobin concentration, and mean corpuscular hemoglobin exhibited compared to the saline group, which confirmed insignificant influence to hepatic and renal functions (Figures 9A,B).

To further explore the potential effect of MCPM NPs on mice, hematoxylin and eosin staining was conducted on the sections from major organs in the control group (saline) and MCPM NP group (days 1, 7, and 21), which showed no appreciable inflammation or lesions (Figure 9C). These experimental results confirmed the non-toxicity of MCPM NPs without laser irradiation. Taken together, these results suggested the negligible toxicity of MCPM NPs *in vivo*.

## Conclusion

In summary, a multifunctional core–shell MSN-Ce6@PDA (Mn) (MCPM NPs) platform was constructed, which were composed of a Ce6-conjugated MSN core and PDA chelated with Mn^2+^ as the shell. The uniqueness of core–shell nanostructures possesses the advantages of both inorganic and organic nanomaterials: non-toxic, with a high-specific surface area, biocompatible, with enhanced MR and fluorescence imaging capacity, with satisfactory photothermal conversion efficiency, and with photodynamic ability. Cancer therapy with MR and fluorescence imaging-guided photothermal and photodynamic combination was achieved *in vitro* and *in vivo*. Furthermore, our MCPM NP nanoplatform would offer more insights into the dual-modal diagnosis of cancer and combination therapy *in vivo*. The multifunctional core–shell platform will shed novel light into combined therapy for breast cancer with bright prospects.

## Data Availability

The original contributions presented in the study are included in the article/Supplementary Files, further inquiries can be directed to the corresponding author.

## References

[B1] Ai F. Ju Q. Zhang X. Chen X. Wang F. Zhu G. (2015). A Core-Shell-Shell Nanoplatform Upconverting Near-Infrared Light at 808 Nm for Luminescence Imaging and Photodynamic Therapy of Cancer. Sci. Rep. 5, 1–11. 10.1038/srep10785 PMC4451683 26035527

[B2] Allison R. R. Cuenca R. Downie G. H. Randall M. E. Bagnato V. S. Sibata C. H. (2005). PD/PDT for Gynecological Disease: A Clinical Review. Photodiagnosis Photodynamic Ther. 2, 51–63. 10.1016/S1572-1000(05)00033-5 25048557

[B3] Amirshaghaghi A. Yan L. Miller J. Daniel Y. Stein J. M. Busch T. M. (2019). Chlorin e6-Coated Superparamagnetic Iron Oxide Nanoparticle (SPION) Nanoclusters as a Theranostic Agent for Dual-Mode Imaging and Photodynamic Therapy. Sci. Rep. 9, 1–9. 10.1038/s41598-019-39036-1 30796251 PMC6385362

[B4] Baldea I. Filip A. G. (2012). Photodynamic Therapy in Melanoma-Aan Update. J. Physiol. Pharmacol. 63, 109–118. 10.1002/9781119084365.ch10 22653896

[B5] Banerjee S. M. MacRobert A. J. Mosse C. A. Periera B. Bown S. G. Keshtgar M. R. S. (2017). Photodynamic Therapy: Inception to Application in Breast Cancer. The Breast 31, 105–113. 10.1016/j.breast.2016.09.016 27833041

[B6] Chen M. Zhang Y. Cui L. Cao Z. Wang Y. Zhang W. (2021). Protonated 2D Carbon Nitride Sensitized with Ce6 as a Smart Metal-free Nanoplatform for Boosted Acute Multimodal Photo-Sono Tumor Inactivation and Long-Term Cancer Immunotherapy. Chem. Eng. J. 422, 130089–138947. 10.1016/j.cej.2021.130089

[B7] Croissant J. G. Fatieiev Y. Almalik A. Khashab N. M. (2018). Mesoporous Silica and Organosilica Nanoparticles: Physical Chemistry, Biosafety, Delivery Strategies, and Biomedical Applications. Adv. Healthc. Mater. 7, 1700831–1700906. 10.1002/adhm.201700831 29193848

[B8] Ebrahimi-Gatkash M. Younesi H. Shahbazi A. Heidari A. (2017). Amino-functionalized Mesoporous MCM-41 Silica as an Efficient Adsorbent for Water Treatment: Batch and Fixed-Bed Column Adsorption of the Nitrate Anion. Appl. Water Sci. 7, 1887–1901. 10.1007/s13201-015-0364-1

[B9] Fan N. Li P. Wu C. Wang X. Zhou Y. Tang B. (2021). ALP-activated Chemiluminescence PDT Nano-Platform for Liver Cancer-specific Theranostics. ACS Appl. Bio Mater. 4, 1740–1748. 10.1021/acsabm.0c01504 35014520

[B10] Goel S. Ferreira C. A. Chen F. Ellison P. A. Siamof C. M. Barnhart T. E. (2018). Activatable Hybrid Nanotheranostics for Tetramodal Imaging and Synergistic Photothermal/Photodynamic Therapy. Adv. Mater. 30, 1704367–1704376. 10.1002/adma.201704367 PMC5805572 29266476

[B11] Guan Q. Zhou L.-L. Li Y.-A. Li W.-Y. Wang S. Song C. (2019). Nanoscale Covalent Organic Framework for Combinatorial Antitumor Photodynamic and Photothermal Therapy. ACS Nano 13, 13304–13316. 10.1021/acsnano.9b06467 31689082

[B44] Guoyun W. Yuanyuan C. Jia S. Qian C. Bowei C. Yuanyuan L. (2020). Chem. Eng. J., 122458–122509.

[B12] Hu C. Zhang Z. Liu S. Liu X. Pang M. (2019). Monodispersed CuSe Sensitized Covalent Organic Framework Photosensitizer with an Enhanced Photodynamic and Photothermal Effect for Cancer Therapy. ACS Appl. Mater. Inter. 11, 23072–23082. 10.1021/acsami.9b08394 31252509

[B13] Hu D. Zhang J. Gao G. Sheng Z. Cui H. Cai L. (2016). Indocyanine Green-Loaded Polydopamine-Reduced Graphene Oxide Nanocomposites with Amplifying Photoacoustic and Photothermal Effects for Cancer Theranostics. Theranostics 6, 1043–1052. 10.7150/thno.14566 27217837 PMC4876628

[B14] Hu J. Zhang X. Wen Z. Tan Y. Huang N. Cheng S. (2016). Asn-Gly-Arg-modified Polydopamine-Coated Nanoparticles for Dual-Targeting Therapy of Brain Glioma in Rats. Oncotarget 7, 73681–73696. 10.18632/oncotarget.12047 27655664 PMC5342007

[B15] Kang Y. Yu X. Fan X. Aodenggerile, Zhao S. Tu C. (2020). Tetramodal Imaging and Synergistic Cancer Radio-Chemotherapy Enabled by Multiple Component-Encapsulated Zeolitic Imidazolate Frameworks. ACS Nano 14, 4336–4351. 10.1021/acsnano.9b09858 32275394

[B16] Lee H. Dellatore S. M. Miller W. M. Messersmith P. B. (2007). Mussel-Inspired Surface Chemistry for Multifunctional Coatings. Science 318, 426–430. 10.1126/science.1147241 17947576 PMC2601629

[B17] Lee T. Y. Cheon Y. K. Shim C. S. Cho Y. D. (2012). Photodynamic Therapy Prolongs Metal Stent Patency in Patients with Unresectable Hilar Cholangiocarcinoma. Wjg 18, 5589–5594. 10.3748/wjg.v18.i39.5589 23112552 PMC3482646

[B18] Li X. Lovell J. F. Yoon J. Chen X. (2020). Clinical Development and Potential of Photothermal and Photodynamic Therapies for Cancer. Nat. Rev. Clin. Oncol. 17, 657–674. 10.1038/s41571-020-0410-2 32699309

[B19] Liang S. Sun C. Yang P. Ma P. a. Huang S. Cheng Z. (2020). Core-shell Structured Upconversion Nanocrystal-Dendrimer Composite as a Carrier for Mitochondria Targeting and Catalase Enhanced Anti-cancer Photodynamic Therapy. Biomaterials 240, 119850–119890. 10.1016/j.biomaterials.2020.119850 32092593

[B20] Liu P. Xie X. Shi X. Peng Y. Ding J. Zhou W. (2019b). Oxygen-Self-Supplying and HIF-1α-Inhibiting Core-Shell Nanosystem for Hypoxia-Resistant Photodynamic Therapy. ACS Appl. Mater. Inter. 11, 48261–48270. 10.1021/acsami.9b18112 31763809

[B21] Liu S. Li W. Dong S. Gai S. Dong Y. Yang D. (2019a). Degradable Calcium Phosphate-Coated Upconversion Nanoparticles for Highly Efficient Chemo-Photodynamic Therapy. ACS Appl. Mater. Inter. 11, 47659–47670. 10.1021/acsami.9b11973 31713407

[B22] Liu X. L. Dong X. Yang S. C. Lai X. Liu H. J. Gao Y. (2021). Biomimetic Liposomal Nanoplatinum for Targeted Cancer Chemophototherapy. Adv. Sci. 8, 2003679–2003694. 10.1002/advs.202003679 PMC8061387 33898179

[B23] Liu Y. Ai K. Liu J. Deng M. He Y. Lu L. (2013). Dopamine-melanin Colloidal Nanospheres: an Efficient Near-Infrared Photothermal Therapeutic Agent for *In Vivo* Cancer Therapy. Adv. Mater. 25, 1353–1359. 10.1002/adma.201204683 23280690

[B24] Lu J. Cai L. Dai Y. Liu Y. Zuo F. Ni C. (2021). Polydopamine‐Based Nanoparticles for Photothermal Therapy/Chemotherapy and Their Synergistic Therapy with Autophagy Inhibitor to Promote Antitumor Treatment. Chem. Rec. 21, 781–796. 10.1002/tcr.202000170 33634962

[B25] Ma L. Zhou Y. Zhang Z. Liu Y. Zhai D. Zhuang H. (2020). Multifunctional Bioactive Nd-Ca-Si Glasses for Fluorescence Thermometry, Photothermal Therapy, and Burn Tissue Repair. Sci. Adv. 6, eabb1311. 10.1126/sciadv.abb1311 32821831 PMC7413731

[B26] Manzano M. Vallet‐Regí M. (2020). Mesoporous Silica Nanoparticles for Drug Delivery. Adv. Funct. Mater. 30, 1902634–1902647. 10.1002/adfm.201902634

[B45] Mengya C. Yule Z. Lifeng C. Ziqi C. Yuwen W. Wei Z. (2021). Chem. Eng. J. 522, 1385-8947.

[B27] Nafiujjaman M. D. Revuri V. Park H. K. Kwon Il. K. Cho K. J. Lee Y. K. (2016). Enhanced Photodynamic Properties of Graphene Quantum Dot Conjugated Ce6 Nanoparticles for Targeted Cancer Therapy and Imaging. Chem. Lett. 45, 997–999. 10.1246/cl.160388

[B28] Ni J. S. Zhang X. Yang G. Kang T. Lin X. Zha M. (2020). A Photoinduced Nonadiabatic Decay‐Guided Molecular Motor Triggers Effective Photothermal Conversion for Cancer Therapy. Angew. Chem. Int. Ed. 59, 11298–11302. 10.1002/anie.202002516 32285540

[B46] Ri W. Huizhen H. L. Wang T. Z. Hou M. He D. G. He X. X. (2020). Chin. Chem. Lett., 209–212.

[B29] Shao D. Zhang F. Chen F. Zheng X. Hu H. Yang C. (2020). Biomimetic Diselenide‐Bridged Mesoporous Organosilica Nanoparticles as an X‐ray‐Responsive Biodegradable Carrier for Chemo‐Immunotherapy. Adv. Mater. 32, 2004385–2004393. 10.1002/adma.202004385 33164250

[B30] Shen D. Yang J. Li X. Zhou L. Zhang R. Li W. (2014). Biphase Stratification Approach to Three-Dimensional Dendritic Biodegradable Mesoporous Silica Nanospheres. Nano Lett. 14, 923–932. 10.1021/nl404316v 24467566

[B31] Sun J.-H. Zhang W. Zhang D.-Y. Shen J. Tan C.-P. Ji L.-N. (2018). Multifunctional mesoporous silica nanoparticles as efficient transporters of doxorubicin and chlorin e6 for chemo-photodynamic combinatorial cancer therapy. J. Biomater. Appl. 32, 1253–1264. 10.1177/0885328218758925 29448866

[B32] Sun W. Luo L. Feng Y. Qiu Y. Shi C. Meng S. (2020). Gadolinium-Rose Bengal Coordination Polymer Nanodots for MR‐/Fluorescence‐Image‐Guided Radiation and Photodynamic Therapy. Adv. Mater. 32, 2000377. 10.1002/adma.202000377 32363649

[B33] Sun X. Sun J. Lv J. Dong B. Liu M. Liu J. (2019). Ce6-C6-TPZ Co-loaded Albumin Nanoparticles for Synergistic Combined PDT-Chemotherapy of Cancer. J. Mater. Chem. B 7, 5797–5807. 10.1039/c9tb01346f 31483422

[B34] Szezerbaty S. K. F. de Oliveira R. F. Pires-Oliveira D. A. A. Soares C. P. Sartori D. Poli-Frederico R. C. (2018). The Effect of Low-Level Laser Therapy (660 Nm) on the Gene Expression Involved in Tissue Repair. Lasers Med. Sci. 33, 315–321. 10.1007/s10103-017-2375-7 29159515

[B35] Wan G. Cheng Y. Song J. Chen Q. Chen B. Liu Y. (2020). Nucleus-targeting Near-Infrared Nanoparticles Based on TAT Peptide-Conjugated IR780 for Photo-Chemotherapy of Breast Cancer. Chem. Eng. J. 380, 122458–1224509. 10.1016/j.cej.2019.122458

[B36] Wang X. Ouyang X. Chen J. Hu Y. Sun X. Yu Z. (2019). Nanoparticulate Photosensitizer Decorated with Hyaluronic Acid for Photodynamic/photothermal Cancer Targeting Therapy. Nanomedicine 14, 151–167. 10.2217/nnm-2018-0204 30511886

[B37] Wang Y. Luo S. Wu Y. Tang P. Liu J. Liu Z. (2020). Highly Penetrable and On-Demand Oxygen Release with Tumor Activity Composite Nanosystem for Photothermal/Photodynamic Synergetic Therapy. ACS Nano 14, 17046–17062. 10.1021/acsnano.0c06415 33290657

[B38] Wei J. Li J. Sun D. Li Q. Ma J. Chen X. (2018). A Novel Theranostic Nanoplatform Based on Pd@Pt-PEG-Ce6 for Enhanced Photodynamic Therapy by Modulating Tumor Hypoxia Microenvironment. Adv. Funct. Mater. 28, 1706310–1706322. 10.1002/adfm.201706310

[B39] Wen L. Hyoju R. Wang P. Shi L. Li C. Li M. (2021). Hydrogen‐Peroxide‐Responsive Protein Biomimetic Nanoparticles for Photothermal‐Photodynamic Combination Therapy of Melanoma. Lasers Surg. Med. 53, 390–399. 10.1002/lsm.23292 32596824

[B40] WuWu R. Wang H. Hai L. Wang T. Hou M. He D. (2020). A Photosensitizer-Loaded Zinc Oxide-Polydopamine Core-Shell Nanotherapeutic Agent for Photodynamic and Photothermal Synergistic Therapy of Cancer Cells. Chin. Chem. Lett. 31, 189–192. 10.1016/j.cclet.2019.05.004

[B41] Yi X. Zhou H. Chao Y. Xiong S. Zhong J. Chai Z. (2020). Bacteria-triggered Tumor-specific Thrombosis to Enable Potent Photothermal Immunotherapy of Cancer. Sci. Adv. 6, eaba3546. 10.1126/sciadv.aba3546 32851163 PMC7428325

[B42] Yu X. Liu X. Wu W. Yang K. Mao R. Ahmad F. (2019). CT/MRI-Guided Synergistic Radiotherapy and X-ray Inducible Photodynamic Therapy Using Tb-Doped Gd-W-Nanoscintillators. Angew. Chem. Int. Ed. 58, 2017–2022. 10.1002/anie.201812272 30589178

[B43] Zhou L. Pi W. Hao M. Li Y. An H. Li Q. (2021). An Injectable and Biodegradable Nano-Photothermal DNA Hydrogel Enhances Penetration and Efficacy of Tumor Therapy. Biomater. Sci. 9, 4904–4921. 10.1039/d1bm00568e 34047319

