# Supplementary material for: Biocompatible Mesoporous Silica–Polydopamine Nanocomplexes as MR/Fluorescence Imaging Agent for Light-Activated Photothermal–Photodynamic Cancer Therapy In Vivo
Source: Front Bioeng Biotechnol. 2021 Nov 9;9:752982. doi: 10.3389/fbioe.2021.752982 (PMC8630682; doi:10.3389/fbioe.2021.752982)
Supplement: Supplementary file 1 [file DataSheet1.docx]

Supplementary Material

## Supplementary Figures


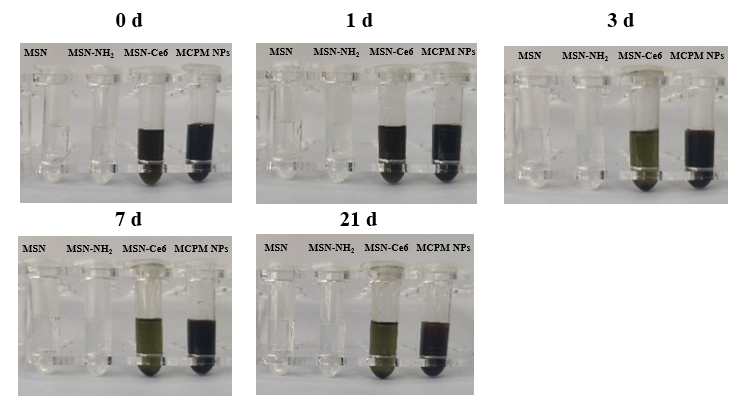


**Supplementary Figure 1.** Photographs of MSN, MSN-NH_2_, MSN-Ce6 and MCPM NPs at days 0, 1, 3, 7 and 21. (From left to right in the photos, four EP tubes are solutions of MSN, MSN-NH_2_, MSN-Ce6 and MCPM NPs)


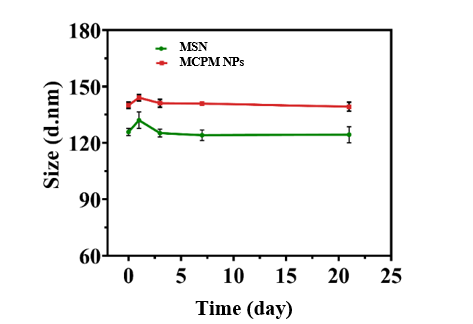


**Supplementary Figure 2.** The hydrodynamic size of MSN and MCPM NPs in a 21-day period. (The green line is solutions of MSN and the red line is solutions of MCPM NPs)


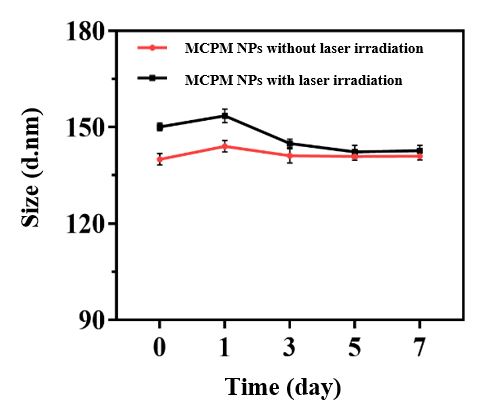


**Supplementary Figure 3.** The hydrodynamic size of MCPM NPs with or without laser irradiation in a 7-day period. (The red line is size of MCPM NPs without laser irradiation and the black line is size of MCPM NPs with laser irradiation)


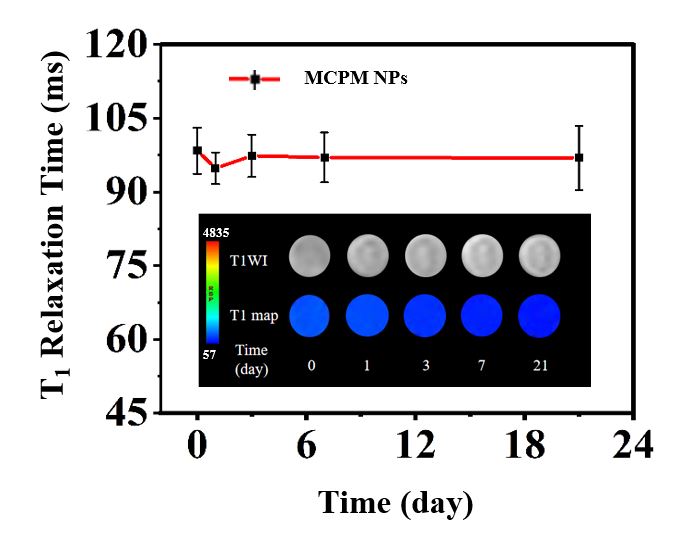


**Supplementary Figure 4**. The changes in T_1_ relaxation time of MCPM NPs in a 21-day period. (The red line is T_1_ relaxation time value of MCPM NPs, the embedded image is the corresponding MRI images)


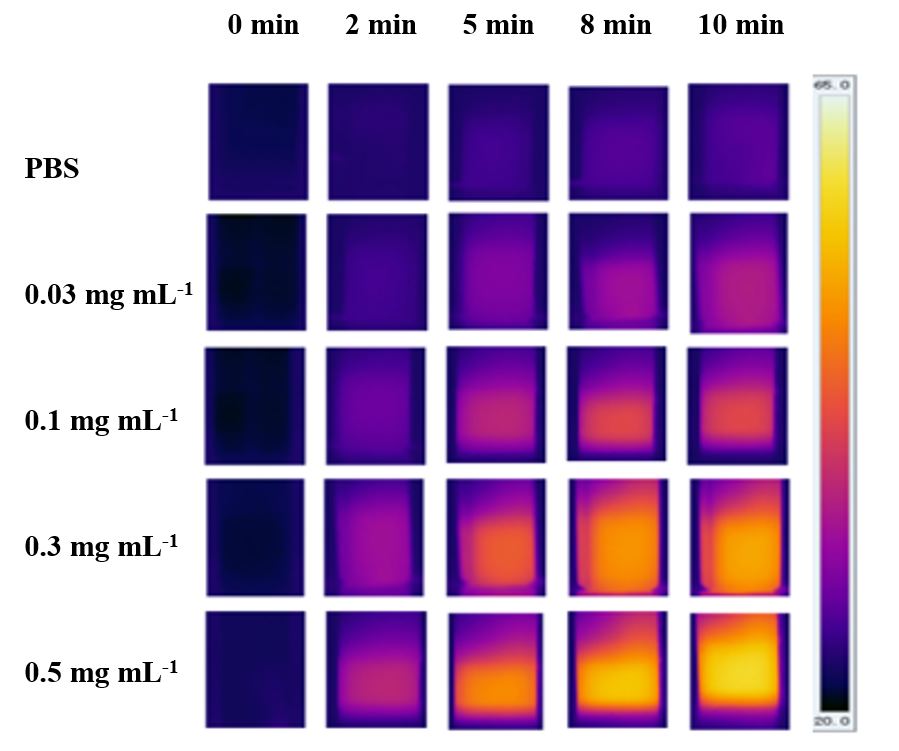


**Supplementary Figure 5**. The infrared thermal images of PBS and different concentrations of MCPM NPs solution (0, 0.03, 0.1, 0.3, 0.5 mg mL^-1^) during 808 nm laser irradiation (0, 2, 5, 8, 10 min).
